# A Specific Nutrient Combination Attenuates the Reduced Expression of PSD-95 in the Proximal Dendrites of Hippocampal Cell Body Layers in a Mouse Model of Phenylketonuria

**DOI:** 10.3390/nu8040185

**Published:** 2016-03-26

**Authors:** Vibeke M. Bruinenberg, Danique van Vliet, Amos Attali, Martijn C. de Wilde, Mirjam Kuhn, Francjan J. van Spronsen, Eddy A. van der Zee

**Affiliations:** 1 Molecular Neurobiology, University of Groningen, 9747 AG Groningen, The Netherlands; 2 Division of Metabolic Diseases, Beatrix Children’s Hospital, University Medical Center Groningen, University of Groningen, 9713 GZ Groningen, The Netherlands; d01.vliet@umcg.nl (D.v.V.); f.j.van.spronsen@umcg.nl (F.J.v.S.); 3 Nutricia Research, Nutricia Advanced Medical Nutrition, 3584 CT Utrecht, The Netherlands; Amos.ATTALI@danone.com (A.A.); martijn.dewilde@nutricia.com (M.C.d.W.); mirjam.kuhn@nutricia.com (M.K.)

**Keywords:** synaptic proteins, Hippocampus, PSD-95, nutrient combination, phenylketonuria

## Abstract

The inherited metabolic disease phenylketonuria (PKU) is characterized by increased concentrations of phenylalanine in the blood and brain, and as a consequence neurotransmitter metabolism, white matter, and synapse functioning are affected. A specific nutrient combination (SNC) has been shown to improve synapse formation, morphology and function. This could become an interesting new nutritional approach for PKU. To assess whether treatment with SNC can affect synapses, we treated PKU mice with SNC or an isocaloric control diet and wild-type (WT) mice with an isocaloric control for 12 weeks, starting at postnatal day 31. Immunostaining for post-synaptic density protein 95 (PSD-95), a post-synaptic density marker, was carried out in the hippocampus, striatum and prefrontal cortex. Compared to WT mice on normal chow without SNC, PKU mice on the isocaloric control showed a significant reduction in PSD-95 expression in the hippocampus, specifically in the granular cell layer of the dentate gyrus, with a similar trend seen in the cornus ammonis 1 (CA1) and cornus ammonis 3 (CA3) pyramidal cell layer. No differences were found in the striatum or prefrontal cortex. PKU mice on a diet supplemented with SNC showed improved expression of PSD-95 in the hippocampus. This study gives the first indication that SNC supplementation has a positive effect on hippocampal synaptic deficits in PKU mice.

## 1. Introduction

The primary defect in the inherited metabolic disease phenylketonuria (PKU) is the disrupted phenylalanine (Phe) metabolism, caused by mutations in the gene encoding for the hepatic enzyme phenylalanine hydroxylase, which normally converts Phe to tyrosine. When no dietary Phe restriction is applied, this causes an increase in blood and brain Phe concentration compared to healthy controls. Although many questions still remain to be answered, a clear correlation between Phe concentration in blood and brain and the cognitive symptoms of PKU has been shown [1,2,3]. Increased Phe concentrations disrupt neurotransmitter metabolism, white matter integrity and synapse functioning in PKU patients and in models of PKU [4,5,6,7,8,9,10,11]. Concerning the latter, a disruption of neuronal connectivity and synaptic morphology became evident in Golgi analyses of both PKU patients and PKU mice, showing a decreased number of spines, width of the synaptic cleft and thickness of the post-synaptic density, indicative of reduced synaptic function [5,6,12]. These observed morphological abnormalities are corroborated by a decrease in proteins associated with synaptic functioning [9,13,14]. As different markers of synaptic functioning have been examined in relation to PKU, the post-synaptic marker post-synaptic density protein 95 (PSD-95) has not. This protein is of interest since it is highly associated with the growth and functioning of dendritic spines and modulates long-term potentiation, a process important for learning and memory [15,16,17]).

Phe-induced neuro-morphological changes are reversible, providing a window of opportunity for interventions even after the expression of symptoms due to high Phe exposure [6,18]. Our study targets these synaptic deficits with a specific nutrient combination (SNC) monitored via the expression of PSD-95. This SNC contains uridine monophosphate (UMP), docosahexaenoic acid (DHA), eicosapentaenoic acid (EPA), choline, phospholipids, folic acid, vitamins B12, B6, C, and E, and selenium. This combination provides rate-limiting nutrients critical for the synthesis of phospholipids, a major component of (synaptic) membranes, and has shown beneficial effects on synapse formation, morphology and function in mouse models of Alzheimer’s disease [19]. Due to the multiple pathways and precursors involved in membrane formation, intervention with single components of this SNC would, very likely, lead to sub-optimal synthesis of phospholipids and therefore limited beneficial effects. Indeed, limited and inconsistent evidence is available for the effect of supplementation with single components of this SNC in PKU patients [20,21,22]. To investigate if SNC can overcome synaptic deficits in PKU, we study here the effect of SNC on the expression of PSD-95 in the hippocampus, striatum and prefrontal cortex in the C57BL/6 PKU mouse model.

## 2. Materials and Methods

### 2.1. Animals and Dietary Intervention

In this study, 31-day-old male and female homozygous C57BL/6 Pah^enu2^ (PKU; gender-balanced groups, *n* = 60) mice and their wild-type (WT; *n* = 10) littermates were fed for 12 weeks with different diets. Mice were bred at the University of Groningen, The Netherlands. At the start of the experiment all mice were housed singly. The genotype of the animals was established by quantitative PCR analysis from DNA extracts from tail tissue [23]. PKU mice were randomly assigned to the following groups: a high-Phe, mid-Phe, or low-Phe diet (Phe contents of 8.8, 6.4, or 4.4 g/kg diet, respectively) either with or without SNC. The Phe contents of 8.8 and 6.4 are in the normal range of standard chow. The 4.4 g/kg of Phe in the food is a slight reduction compared to commercially available standard chows but still contains the minimal nutritional requirement for laboratory animals [24]. The different Phe concentrations in food resulted in the following Phe concentrations in blood: WT control; 49.6 ± 5.9, PKU 8.8; 1841.3 ± 305.4, PKU 6.4; 1413.8 ± 199.8, and PKU 4.4; 1065 ± 150.4 (mean ± standard deviation). WT control mice received a high-Phe diet without SNC. A WT control group on this same diet with SNC was not included because potential beneficial effects are considered irrelevant to elucidate the hypothesized mode of action of SNC in the PKU model. All components were in accordance with the minimal nutritional requirement for laboratory animals [24]. This study was approved by the ethical committee of the University of Groningen, The Netherlands.

### 2.2. Tissue Preparation

After 12 weeks of dietary treatment, all animals were euthanized via a single intraperitoneal injection of pentobarbital. Blood was collected via heart punction and animals were transcardially perfused with 4% paraformaldehyde (PFA; in 0.1 M phosphate buffer (PB); pH 7.4). Brains were post-fixed for 24 h and subsequently rinsed with 0.01 M PB. After exposing the brains to 30% buffered sucrose, they were snap-frozen with liquid nitrogen and stored at −80 °C.

### 2.3. Immunohistochemistry

**Figure 1:**
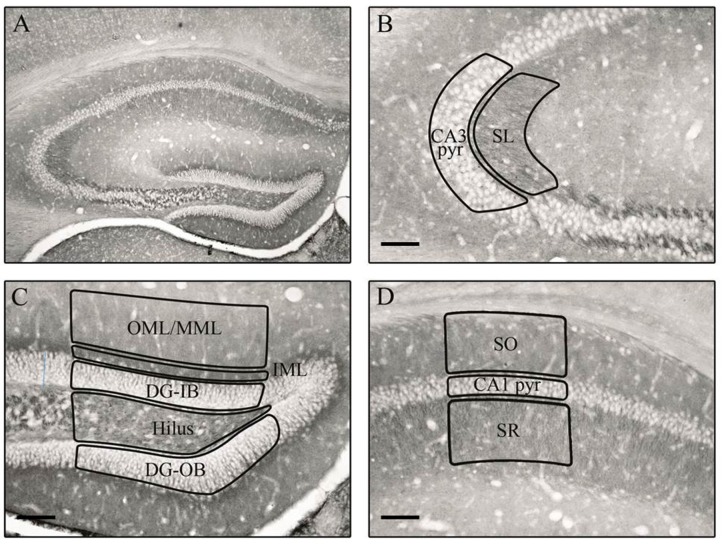

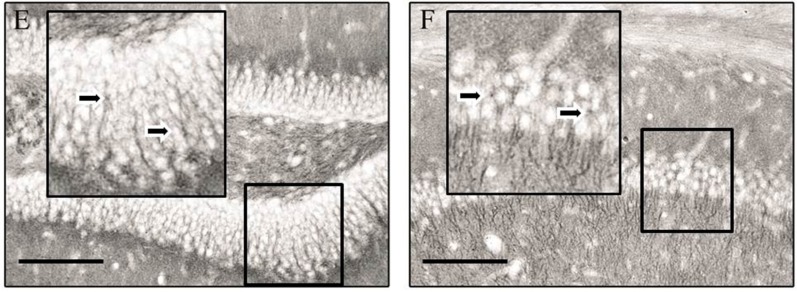
PSD-95 expression in the C57BL/6 PKU mouse. (**A**) An overview picture of the PSD-95 immunostaining; The following 10 areas of interest were measured: (**B**) CA3: stratum lucidum (SL), CA3 pyramidal cell layer (CA3 pyr); (**C**) DG: outer and middle molecular layer (OML/MML), the inner molecular layer (IML), inner (DG-IB) and outer blade (DG-OB) of the dentate gyrus, hilus; (**D**) CA1: stratum oriens (SO), CA1 pyramidal cell layer (CA1 pyr) and the stratum radiatum (SR); (**E**) a detailed picture of the granular layers present in the DG; (**F**) a detailed picture of the CA1 pyramidal cell layer. Arrows indicate clear staining within the granular layer. The size bar indicates 100 µm. The detail pictures within (**E**) and (**F**) are digitally enlarged.

Coronal brain sections (20 µm thick) were processed for immunohistochemical analysis of the post-synaptic marker PSD-95 with a free-floating technique according to the following steps: (1) incubation with 0.3% H_2_O_2_ for 30 min; (2) incubation with 1:1000 monoclonal mouse anti-PSD-95, Millipore, MABN68, 1% normal goat serum (NGS) and 0.5% Triton-X for 2 h in a water bath at 37 °C, 24 h at room temperature and subsequent storage for 48 h at 4 °C; (3) incubation with secondary antibody solution (1:500 Biotin-SP-conjugated affiniPure Goat-anti-Mouse, Jackson, code: 115-0.65-166 Lot# 110630, 1% NGS and 0.5% Triton-X) for 2 h at room temperature (RT); (4) incubation with 1:400 AB complex, Vectastain PK-6100 standard in TBS for 2 h at RT; (5) color development was initiated by the introduction of 100 µL 0.1% H_2_O_2_ to the 3,3′-diaminobenzidine (DAB; 7 mg/15 mL) solution. The sections were rinsed multiple times with Tris buffered Saline (pH 7.4) between the above-described steps. The specificity of the primary antibody was tested with omitting the primary antibody in the protocol of the staining, which resulted in the absence of detectable immunostaining, and Western blot, which showed a band at 95 kDa. The optical density (OD) of the staining was measured with a Quantimet 550 image analysis system (Leica, Cambridge, UK) as has been used before [25,26]. In the hippocampus, 10 regions of interest were measured between bregma coordinates −1.34 and −1.82 mm (see Figure 1 for delineation and abbreviations). In the striatum, the mean OD of three fixed areas located in the caudoputamen were calculated (between bregma coordinates 1.18 and 0.14 mm). In addition, the infralimbic and prelimbic areas of the prefrontal cortex were measured between the bregma coordinates 1.98 and 1.54 mm. The OD of these regions was corrected for background staining by subtracting the OD of the corpus callosum. Both hemispheres of three sections of each individual were measured. Due to frozen artifacts, three animals were discarded: one animal from the high-Phe without SNC group and two animals from the low-Phe without SNC group.

### 2.4. Statistical Analysis

The distribution of all parameters was checked with the Shapiro-Wilk normality test. Normally distributed data were tested with a one-way ANOVA with the Bonferroni test as a *post hoc* test. The Kruskal-Wallis test was used for non-parametric data with the Mann-Whitney U test as a *post hoc* test. All statistical analyses were performed with the Statistical Package for Social Sciences (SPSS) (SPSS Inc. SPSS for Windows, Version 16.0. Chicago, IL, USA).

## 3. Results

Body weight and blood Phe concentrations were measured to monitor the dietary treatment. The absolute Phe intake, corrected for body weight, food intake, and the measured Phe in the food, confirmed that Phe intake was significantly different between the different Phe content groups but not between the different Phe content groups and their corresponding SNC groups (one-way ANOVA: *df* = 6; *F* = 64.181, *post hoc* Bonferroni analysis: High-Phe-Mid-Phe; *p* = 0.000 High-Phe-Low-Phe; *p* = 0.000, Mid-Phe-Low-Phe; *p* = 0.000, High-Phe with and without; *p* = 0.298, Mid-Phe with and without; *p* = 1.000, Low-Phe with and without; *p* = 1.000).

Furthermore, as it is clinically relevant, both males and females were used in this study. It is known from the literature that hippocampal spine density is affected by the estrous cycle and hormones associated with this cycle [27]. However, we did not find significant differences between males and females in the PSD-95 OD, and therefore pooled values from males and females.

**Figure 2:**
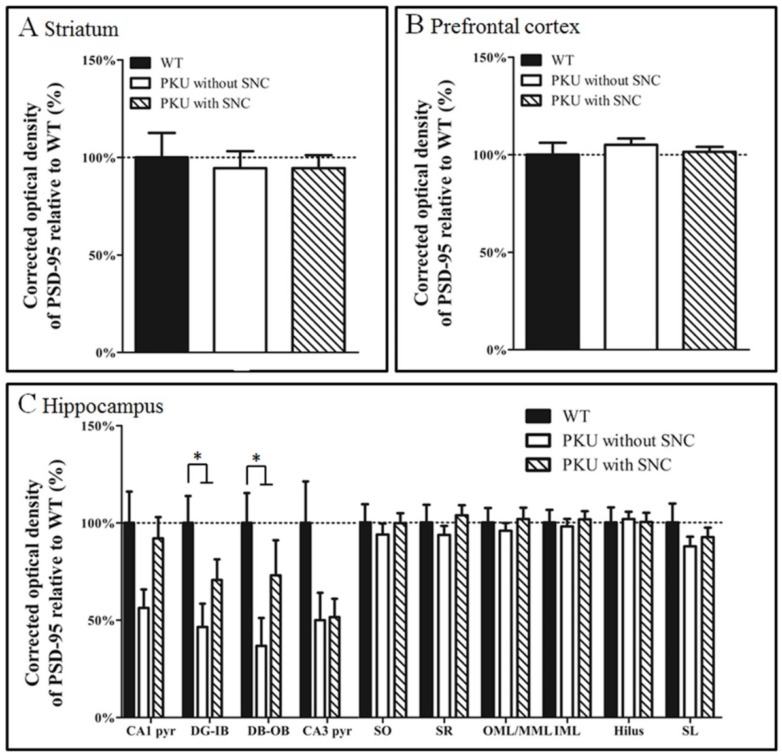
PSD-95 expression is reduced specifically in the hippocampus of the PKU mouse model. (**A**) No significant differences are found between the three groups in striatum (Kruskal-Wallis: *p* = 0.830); (**B**) No significant differences are found between the three groups in the prefrontal cortex (Kruskal-Wallis: *p* = 0.930); (**C**) Compared to WT mice on normal chow without SNC, PKU mice on the isocaloric control showed a significant reduction in PSD-95 expression in the hippocampus, specifically in the granular cell layer of the dentate gyrus (Kruskal-Wallis: DG-IB *p* = 0.009, DG-OB *p* = 0.008), with a similar trend in the CA1 and CA3 pyramidal cell layer (Kruskal-Wallis: CA1 pyr *p* = 0.053, CA3 pyr *p* = 0.064). A significant difference was found between the WT group and both PKU groups for the DG-IB and DG-OB (WT compared to PKU without SNC: Mann-Whitney U test DG-IB; *p* = 0.005, DG-OB; *p* = 0.005. WT compared to PKU with SNC: Mann-Whitney U test DG-IB; *p* = 0.031, DG-OB; *p* = 0.026) (arrow bars depict SEM).

Non-parametric tests were used to examine the PSD-95 immunoreactivity differences. No significant differences were found between the Phe-content groups of PKU mice for all brain regions studied. Therefore, all PKU mice from the different Phe groups were pooled into two groups: those with and without SNC supplementation (final groups: WT, PKU with and PKU without SNC). The immunoreactivity of these three groups are depicted in Figure 2. In the hippocampus, a significant difference was found within the inner blade and outer blade of the dentate gyrus (Kruskal-Wallis: inner blade of dentate gyrus (DG-IB); *p* = 0.009, outer blade of dentate gyrus (DG-OB); *p* = 0.008). *Post hoc* testing revealed that PSD-95 OD in the DG-IB and DG-OB was significantly decreased in PKU mice fed with diets without SNC compared to WT by 54% and 64%, respectively (Mann-Whitney U test DG-IB; *p* = 0.005, DG-OB; *p* = 0.005). Although these significant differences were still present in DG-IB and DG-OB between the WT and PKU mice fed with diets with SNC, the decrease was considerably less: 29% and 27%, respectively (Mann-Whitney U test DG-IB; *p* = 0.031, DG-OB; *p* = 0.026). A clear trend was observed in the cornus ammonis 1 (CA1) and cornus ammonis 3 (CA3) cell layers (Kruskal-Wallis: CA1; *p* = 0.053, CA3; *p* = 0.064), where PKU mice on diets without SNC showed a reduction in PSD-95 OD of 44% in the CA1 and 51% in the CA3 compared to WT mice. In the CA1, this difference was attenuated for the PKU mice fed with diets with SNC, as the difference between this group and WT mice was only 8%. In the striatum and prefrontal cortex, no significant differences were found between the groups (Kruskal-Wallis: *p* = 0.830, *p* = 0.930, respectively); no PKU PSD-95 expression deficit was present in PKU mice, and no change was induced by SNC.

## 4. Discussion

To the best of our knowledge, this is the first report of reduced hippocampal PSD-95 expression in the PKU mouse model. The most important finding of our study is that SNC supplementation seems to dampen PKU PSD-95 OD deficits towards WT values in specific areas of the hippocampus. Therefore, this study indicates that SNC supplementation could have a positive effect on synaptic functioning by lessening the reduced expression of PSD-95 in the hippocampus of C57BL/6 PKU mice in the regions affected in PKU. This positive effect was most clear in the dendrites of the DG granular cells at the level of the cell layer, and to a lesser extent in CA1 pyramidal cell dendrites at the level of the cell layer.

PSD-95 is a scaffolding protein which is post-synaptically present in glutamatergic and serotonergic synapses [15,28]. In general, PSD-95 is implicated in post-synaptic plasticity and maturation of excitatory synapses via the interchange of α-amino-3-hydroxy-5-methyl-4-isoxazolepropionic acid (AMPA) and N-methyl-D-aspartate (NMDA) receptors in the post-synaptic compartment [15,29,30,31]. The most marked reduction in PSD-95 in the PKU hippocampus was found in the DG granular layer, and was somewhat less prominent in the CA1 and CA3 regions. In all cases, the reduction was limited to the most proximal part of the dendrites. At present, it is unclear which hippocampal input circuitry anatomically best matches the affected terminal fields. The hippocampus receives input from various sources, which deviate in their terminal fields. For example, part of the input from the entorhinal cortex terminates on the proximal dendrites of DG granular cells and CA3 pyramidal cells, and the Schaffer collaterals originating from CA3 pyramidal neurons project on the CA1 proximal dendrites [32,33]. A reduction of PSD-95 in PKU mice, specifically within the proximal dendrites, could suggest weakening of synaptic connectivity in these neuronal circuits which could negatively impact learning and memory. The supplementation with SNC attenuates the PKU-specific reduction in hippocampal PSD-95 expression towards WT levels. Although the effect was not statistically significant for all regions, SNC treatment seems to particularly affect the CA1, DG-IB, and DG-OB. Apparently, certain cellular properties of these regions are more susceptible to the effect of SNC. Alternatively, the duration of the treatment was not sufficient to have a strong positive effect in all hippocampal regions.

The relation between PSD-95 and the glutamatergic AMPA and NMDA receptors suggests that the PKU-specific reduction in PSD-95 could specifically affect these receptors. However, the literature concerning this topic is somewhat contradictory. Martynyuk *et al.* [34] found a significant increase in the Glu1 and Glu2/3 subunits of AMPA receptors and a total increase in NMDA receptor densities, a suggested compensatory mechanism for the acute suppressive effect of high Phe concentrations on glutamatergic synaptic transmission [32]. Despite the up-regulation in post-synaptic glutamate receptors, Martynyuk *et al.* [34] also report preliminary data indicating that the functional activity of glutamatergic synaptic transmission in the PKU brain is still reduced. Although they do not specify to what degree, it is possible to envisage that the reduction in function is associated with the reduction in PSD-95 found in our study.

In consensus with the literature, this study shows reduced levels of a protein associated with synaptic functioning in PKU mice [9,13,14]. In contrast, recent literature showed an increase in synapse number and an increase in the post-synaptic markers synaptopodin and spinophilin in specific regions of the hippocampus (striatum radiatum CA1 and stratum lucidum CA3) which could suggest that this results in an increase in PSD-95 in the same regions [14]. However, our study did not show significant differences between PKU and WT individuals in the same hippocampal subregions. Horling and colleagues [14] found a specific upregulation of thin and branched spines, and the post-synaptic markers associated with the cytoskeleton, suggesting that the less mature spines are affected. These immature spines, which are not fully engaged in synaptic activity, could contain less PSD-95 compared to the types that were not affected, e.g., the mushroom type. Hence, our data suggest that the increase in the number of spines and post-synaptic markers found by Horling and colleagues does not come with an overall increase in PSD-95 expression.

To conclude, this study shows that SNC supplementation could have a positive effect on PSD-95 expression in the specific hippocampal subregions affected in C57BL/6 PKU mice. The examination of additional pre-and post-synaptic markers and functional outcomes, e.g., executive functioning, will be key to the subsequent more extensive investigation of synaptic dysfunction in PKU mice and the beneficial effects of SNC supplementation.
